# Growing vegetables on frozen ground

**DOI:** 10.2471/BLT.22.020122

**Published:** 2022-01-01

## Abstract

The Russian Federation is promoting year-round greenhouse production of vegetables to help change the population’s eating habits. Andrei Shukshin reports.

If it were not for the greenhouse, the 350 000 residents of Yakutsk would be a long way from the nearest fresh tomato.

The capital of the Yakutia region of the Russian Federation about 450 kilometres from the Arctic Circle, Yakutsk has an average annual temperature of −8.8° C, making it the coldest city in the world.

“Until a few years ago we had to make do with fruit and vegetables that were shipped in from as far away as China,” says 30-year-old local resident Irina Makarova (name changed at her request). “They had often been in transit for at least a week and weren’t very fresh.”

The bulk of those products came from Novosibirsk, a major regional hub for fruit and vegetables, 4880 kilometres away by road. The source started to change in 2016 when the Yakutsk city administration and Yakutsk-based commercial bank, Almazergienbank, signed an investment agreement with Japan’s Hokkaido Corporation for the construction of greenhouses on a site 20 kilometres to the north of the city.

“We were happy to work with the Japanese,” says Yevdokia Yevsikova, first deputy chairwoman of the Yakutsk city legislature. “They had a lot of experience building greenhouses in cold climates and were eager to see if their equipment was robust enough to cope with Yakutsk temperatures.”

According to Yevsikova, the driving force behind Sayuri, as the greenhouse project is known (the name means “small lily” in Japanese) was Aysen Nikolayev, the former mayor of Yakutsk and now Head of the Sakha Republic. One of Nikolayev’s principal concerns was ensuring that the local population got access to fresh vegetables.

Built in 2016, the first greenhouse covered 1000 square metres, was assembled from components imported from Japan and ran on electricity drawn from Yakutsk’s coal-powered grid.

“Building a piece of the tropics on permafrost is not easy,” says Sayuri’s chief agronomist Timir Ivanov, listing challenges that include annual temperature variations from − 60° C in winter to 40° C in summer. “The temperature inside the greenhouse must be a constant 24° C with 100% humidity, so the build-up of condensation was a real problem,” Ivanov explains.

To get round it, Sayuri’s engineers installed double glazing made from a transparent film sandwiching a layer of warm air. According to Ivanov, the film, which is supplied by Asahi Glass, a Japanese corporation, has non-stick and self-cleaning properties and is expected to last over a decade in local conditions.

“Building a piece of the tropics on permafrost is not easy.”

Timir Ivanov

Starting with lettuce, Sayuri has gone on to produce cucumbers, tomatoes and strawberries. Produced hydroponically (using mineral nutrient solutions instead of soil), the plants can be grown all year round.

According to Yevsikova, in 2020, the complex yielded over 617 tonnes of vegetables, and as of late November, looked set to double that amount in 2021. The jump in output reflects in part a significant increase in capacity. Completed in December, the complex now covers 3.3 hectares, making it the biggest such project built on permafrost in the Russian Federation.

But not the only one. In fact, similar projects are being developed elsewhere in the country, with 30 greenhouses being built or upgraded adding to the 50 already established. Most are public/private initiatives, such as the Sayuri project, while some have started out as private and have then attracted state support in various forms including subsidized loans.

This is true, for example of the project in the Anadyr industrial park in Chukotka, the capital of the country’s easternmost region, where a Soviet-era metal hangar was converted into a greenhouse by local entrepreneurs, initially producing just herbs. After securing a state-subsidized loan, the project added two modern greenhouses and now supplies the town of 15 000 inhabitants with lettuce and cucumbers as well as flowers.

On a visit to Anadyr in August 2020, Russian Prime Minister Mikhail Mishustin was so impressed with his walk along rows of cucumbers 200 kilometres from the Arctic Circle that he ordered it to be used as a blueprint for complexes elsewhere. Three months later, he announced that the federal government would start reimbursing investors for 20% of capital costs of winter greenhouses (permanent structures designed to grow fruit and vegetables year-round) in the far east regions of the country. “Developing greenhouse vegetable production is important both from the point of view of food security and for people to have access to fresh vegetables all year round,” he said at the announcement.

According to the latest agricultural ministry data, between January and November this year over 1.1 million tonnes of vegetables and greens were harvested in winter greenhouses in the Russian Federation, up 3.6% on the same period of 2020. The output included 651 thousand tonnes of cucumbers and 458 thousand tonnes of tomatoes. Over the last 10 years the total area of greenhouses has increased by 40% and output more than doubled from 615 thousand tonnes in 2013 to an expected 1.4 million tonnes in 2021.

All this activity reflects a push by the federal government to ensure food security while also encouraging Russians to adopt healthier diets as part of efforts to reduce the noncommunicable disease burden related to overweight and obesity.

According to Rospotrebnadzor, the federal agency responsible for monitoring and protecting citizens’ health, 47.6% of Russian men are overweight and 19% obese, while 35.6% of women are overweight and 27.6% are obese. The federal government would like to see those numbers come down and in 2019 rolled out the “Strengthening Public Health” project, one pillar of which is healthy eating.

The extent to which the project is having an impact on people’s eating habits is unclear, although recent surveys conducted by Rospotrebnadzor’s research partner, the Federal Research Center for Nutrition, Biotechnology and Food Safety, indicate a gradual shift towards healthier foods with increased intakes of dairy products, vegetables and fruit.

The latest large-scale survey carried out by the Federal Service for State Statistics (Rosstat) in 2018 (before the Strengthening Public Health project began) indicated that Russian women on average consumed 78.1 kg of vegetables, down from the 98.5 kg reported in 2013 (the year of the previous survey), and men consumed 97.2 kg compared with 116.7 kg in 2013.

In Yakutia, average consumption of vegetables may be even lower. “Actual annual vegetable intake in Yakutia is 70-77 kilos per capita, including about seven kilos of cucumbers and five of tomatoes,” says Dr Alevtina Everstova, head doctor at Multiprofile clinic No 1 in Yakutsk. “That is significantly below WHO’s recommendation of around 146 kilos of fruit and vegetables per year.”

“[The complex] is giving us something money can’t buy – better health for our children.”

Alevtina Everstova

According to Yevsikova, one reason for the relatively low fruit and vegetable consumption is local food culture. “Hostile weather conditions and availability of produce have been key in shaping local diets which are high in carbohydrate, meat and fish and low in vegetables and fruit,” she says.

Can Sayuri change that culture? With a projected output of around 1500 tonnes of fruit and vegetables, primarily cucumbers and tomatoes, the complex will make only a modest difference to a region that annually consumes about 36 000 tonnes. But more greenhouses may be able to make a difference, particularly if the produce they generate is directed towards a group that may be less wedded to local culinary traditions – i.e. children.

That is the hope of the Yakutsk authorities who are directing most of the produce grown in Sayuri to their youngest citizens. There is a small shop at the complex and some deliveries go to merchants across the city, but the bulk is sent to local schools and kindergartens, fully covering their nutritional requirements, according to Everstova.

This is in line with the policy articulated at the federal level. In March 2020, President Vladimir Putin signed into law amendments to the Federal Law on the Quality and Safety of Food which, for the first time, introduced into Russian legislation the concept of "healthy eating", establishing the principles of healthy diets as well as requirements for meals to be provided to certain population groups, including children, the elderly, hospital patients and others.

Some Yakutsk residents have raised questions about the cost of the Sayuri project to the local administration. According to Ivanov, Sayuri is doing everything it can to bring those costs down. “The company switched to domestic greenhouse frame suppliers and is using local workforce to install and operate all but the most expensive and insurance-covered equipment. The switch to gas also brought down costs,” he says.

For Everstova, the extra expenditure is worth it. “We hear some people say that the Sayuri complex is an expensive endeavour,” she says, “but it is giving us something money can’t buy – better health for our children.”

**Figure:**
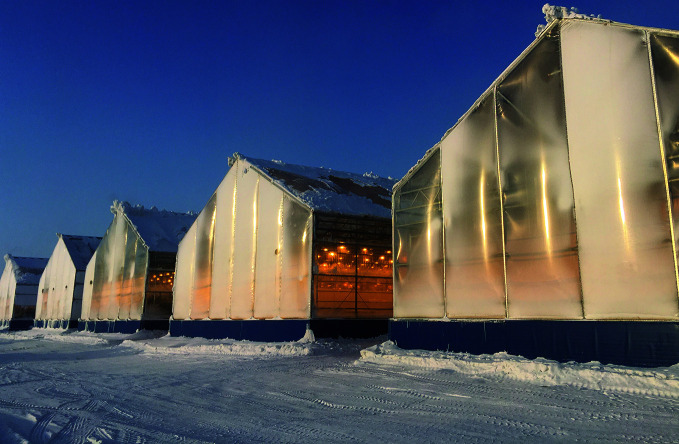
Double-glazed greenhouses in the Sayuri complex

Courtesy of Sayuri

**Figure:**
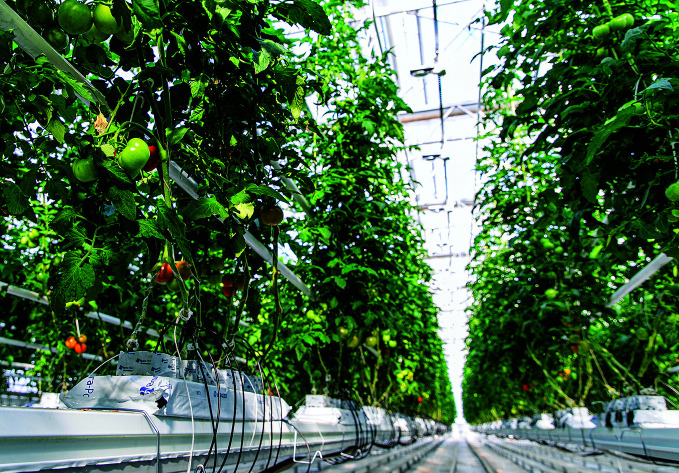
Tomatoes ripening in a Sayuri greenhouse

Courtesy of Sayuri

